# Jaundice in the New-Born

**Published:** 1907-10-26

**Authors:** 


### Diseases of Children.

### JAUNDICE IN THE NEW-BORN.

The common form of jaundice in the new-born varies from a pale to an intense yellowness, which appears usually on the third to the fifth day of life, increases in depth for two or three days, and then slowly disappears. In the more severe cases bile pigment and salts are found in the urine, and the colour of the stools is affected; occasionally nutrition is impaired. Few cases last more than a week or two: rarely the jaundice persists for as many months, though it does not make the child ill, and ends in complete recovery. No special treatment is needed. Small doses of grey powder, calomel, or sodium bicarbonate are beneficial. A fatal result may ensue from some associated condition, and in such cases almost all the organs except the liver are bile stained. Extreme viscidity of the bile, retention of bile, and venous stasis in the liver have been found. A tendency to the secretion of viscid bile may be a family peculiarity, and explain such instances as that of a family in which nine infants died under one month of age from jaundice, and a tenth died at six years of age from the same cause (Underwood). A similar instance has come under our notice, and others have been recorded.

The jaundice is due to re-absorption of bile, which is formed in increased quantity from the disintegrated red corpuscles at and after birth, the bile being retained in the liver through obstruction to its free circulation. This retardation in the liver is partly due to venous congestion, and partly to viscidity of the bile and its secretion at a low pressure. Hence, jaundice is most common in feeble infants and those born asphyxiated. Further evidence of the tendency to stagnation of bile in the liver is the fact that biliary concretions in this organ are more common in infancy than at any other period of childhood. It may even occur *in utero*, causing intense jaundice at birth or shortly after, with calculous obstruction of the ducts. Another possible explanation of the family cases is that a catarrhal cholangitis is set up by some toxin transmitted from the mother.

Such an explanation has been put forward for those more serious cases described under the name of *Congenital Obliteration of the Bile Ducts.* Varied anatomical defects are included under this name. Thus the duct may be only obliterated at its entrance into the duodenum, or the gall bladder and all the bile ducts may be absent or obliterated, or there may be intermediate degrees of absence or obliteration. Should the cystic duct alone be affected there may be no symptoms. Jaundice is present in about one-fourth of these cases from birth, and in the rest comes on in two or three days, or even not for a fortnight. It steadily increases and becomes very intense. The stools are pale, white, like thick milk, and offensive; the urine becomes greenish brown. Flatulent distension results from decomposition of intestinal contents. The liver is enlarged, and may even reach the anterior superior spine of the ilium. Subcutaneous hæmorrhages and bleeding, from the umbilicus and mucous membranes, are common. The babe rapidly wastes, develops ascites and œdema of the extremities, and dies in about six to eight weeks from asthenia or convulsions. Occasionally life has been prolonged for eight or nine months. Some infants succumb in a few days. It is on the results of post-mortem examination that our knowledge of this condition is mainly established. Hence arises the name of "congenital hepatic cirrhosis with obliteration of the bile ducts," for the liver changes are the most characteristic feature. The liver exhibits a biliary or monolobular cirrhosis, such as can be produced experimentally in dogs by ligature of the bile ducts. Rolleston, however, describes the cirrhosis as partly ordinary portal cirrhosis and partly of the biliary type.

For many years it has been customary to explain the affection as the result of a primary malformation, the changes in the liver being secondary. Rolleston's view that it is due to a primary descending cholangitis, which causes the cirrhosis and secondary obliteration of the ducts, is the more probable, though it does not explain those instances of malformation limited to the duodenal end of the common duct. Recently Emanuel has recorded a case (*British Medical Journal*, 1907, ii. 385) which strongly supports Rolleston's explanation. In this child the bile ducts were absent, the gall bladder rudimentary, the liver cirrhotic and of the biliary type, the spleen fibrous, and the pancreas also cirrhotic in relation to its ducts, similarly but to a less marked extent than the liver.

Rolleston has also reported (*British Medical Journal*, 1907, ii. 947) a parallel case as one of congenital syphilitic obstruction of the common bile duct. Death took place at three weeks from jaundice, melæna and wasting. The anatomical changes were pericholangitis fibrosa of the hepatic and common ducts, obstruction of the common duct half an inch from the duodenum by granulation tissue, pericellular and periportal cirrhosis microscopically, and diffuse inter-acineus fibrosis of the pancreas.

Such a combination of changes can be explained as follows:—Some toxin is transmitted from the mother to the fœtus. The liver receives most of the poison through the portal vein, and a further dose through the hepatic vein, *via* the general circulation and the ductus venosus. The toxin reaches the spleen and pancreas also through the general circulation. If the toxin is transmitted early in fœtal life the hepatic cirrhosis and cholangitis may cause arrest of development of ducts and gall bladder. Later it may cause obliteration. Some of the cases may be purely developmental, notably those in which the obliteration is at the duodenal end of the duct. Probably the syphilitic poison is a frequent cause.

Treatment naturally resolves itself into attention to the health of the mother during pregnancy, and even before conception. The differential diagnosis between the simple icterus neonatorum and the fatal cirrhotic cases is sometimes extremely difficult, and quite impossible in early stages.

